# A protein kinase C α and β inhibitor blunts hyperphagia to halt renal function decline and reduces adiposity in a rat model of obesity-driven type 2 diabetes

**DOI:** 10.1038/s41598-023-43759-7

**Published:** 2023-10-07

**Authors:** Ju Wang, Agustin Casimiro-Garcia, Bryce G. Johnson, Jennifer Duffen, Michael Cain, Leigh Savary, Stephen Wang, Prashant Nambiar, Matthew Lech, Shanrong Zhao, Li Xi, Yutian Zhan, Jennifer Olson, James A. Stejskal, Hank Lin, Baohong Zhang, Robert V. Martinez, Katherine Masek-Hammerman, Franklin J. Schlerman, Ken Dower

**Affiliations:** 1 Inflammation and Immunology, Pfizer Worldwide Research and Development, Cambridge, MA USA; 2 Medicine Design, Pfizer Worldwide Research and Development, Cambridge, MA USA; 3 Present Address: Mediar Therapeutics, Boston, MA USA; 4 Present Address: Instem Life Science Systems Ltd, Mount Ida College, South Hadley, MA USA; 5 Pharmacokinetics and Drug Metabolism, Pfizer Worldwide Research and Development, Cambridge, MA USA; 6 Present Address: Novartis Gene Therapies, Novartis Institute for Biomedical Research, Cambridge, MA USA; 7 Drug Safety Research and Development, Pfizer Worldwide Research and Development, Cambridge, MA USA; 8 Present Address: Strand Therapeutics, Cambridge, MA USA; 9 Clinical Genetics and Bioinformatics, Pfizer Worldwide Research and Development, Cambridge, MA USA; 10 Present Address: Amunix Pharmaceuticals, San Francisco, CA USA; 11 Early Clinical Development, Pfizer Worldwide Research and Development, Cambridge, MA USA; 12 Drug Safety Research and Development, Pfizer Worldwide Research and Development, Groton, CT USA; 13 Present Address: Charles River Laboratories, Shrewsbury, MA USA; 14 Present Address: Sunovion Pharmaceuticals Inc., Marlborough, MA USA; 15 Present Address: Data Sciences, Biogen, Cambridge, MA USA; 16 Present Address: Center for Technological Innovation, Pfizer Worldwide Research and Development, San Francisco, CA USA

**Keywords:** Drug discovery, Kidney, Metabolic diseases

## Abstract

Type 2 diabetes (T2D) and its complications can have debilitating, sometimes fatal consequences for afflicted individuals. The disease can be difficult to control, and therapeutic strategies to prevent T2D-induced tissue and organ damage are needed. Here we describe the results of administering a potent and selective inhibitor of Protein Kinase C (PKC) family members PKCα and PKCβ, Cmpd 1, in the ZSF1 obese rat model of hyperphagia-induced, obesity-driven T2D. Although our initial intent was to evaluate the effect of PKCα/β inhibition on renal damage in this model setting, Cmpd 1 unexpectedly caused a marked reduction in the hyperphagic response of ZSF1 obese animals. This halted renal function decline but did so indirectly and indistinguishably from a pair feeding comparator group. However, above and beyond this food intake effect, Cmpd 1 lowered overall animal body weights, reduced liver vacuolation, and reduced inguinal adipose tissue (iWAT) mass, inflammation, and adipocyte size. Taken together, Cmpd 1 had strong effects on multiple disease parameters in this obesity-driven rodent model of T2D. Further evaluation for potential translation of PKCα/β inhibition to T2D and obesity in humans is warranted.

## Introduction

Diabetes affects an estimated 170 million people worldwide, posing enormous humanitarian and economic challenges^1–3^. Complications from diabetes account for an estimated 1 in every 8 dollars spent on medical care in the United States^1^. The disease can be difficult to control, and large numbers of individuals succumb to diabetes-induced tissue damage that can culminate in limb amputation, blindness, end-stage renal disease (ESRD), and death. Indeed, the Centers of Disease Control estimates that diabetes is the 7th leading cause of death in the United States^4^. A clear and present need exists for therapeutic strategies for afflicted individuals, in particular given the increasing incidence in obesity which is a significant risk factor^2,5^.

A serious comorbidity of diabetes is diabetic nephropathy (DN), which is characterized by cellular and structural aberrations in the kidney^6^. DN is the leading cause of ESRD in the United States and is also a major risk factor for heart attack and stroke^7,8^. Several signaling molecules and pathways have been implicated in DN progression (reviewed in^8,9^). Among these are the PKC family of serine-threonine kinases. PKCs consists of conventional family members α (alpha), β (beta, with β1 and β2 splice variants), and γ (gamma); novel family members δ (delta), ε (epsilon), θ (theta) and η (eta); and atypical family members ζ (zeta) and ι/λ (iota in humans, referred to as lambda in rodents). Conventional and novel members are activated directly by diacylglycerol (DAG), the levels of which increase in diabetes due to de novo synthesis^10,11^. They are also indirectly activated by oxidative stress and advanced glycosylation end products (AGEs), which increase during diabetic tissue injury^12,13^. Because the activation of PKCs themselves can result in the production of reactive oxygen species (ROS), their activation has been postulated to lead to a destructive feed-forward loop culminating in cell and tissue damage in the diabetic setting^14^.

PKCα and PKCβ have been implicated in human and preclinical DN through elevated expression (PKC α and β) and genetic association (PKCβ)^15–22^. Studies using single and double knockout mice in models of streptozotocin (STZ)-induced type 1 diabetes (T1D) indicate non-redundant roles for these two PKCs in promoting DN: namely, PKCα in the loss of podocyte integrity and increased permeability of the glomerular filtration barrier, and PKCβ in renal cell hypertrophy and fibrosis^23–28^. Consistent with this, administration of staurosporine-based PKC inhibitors such as Ruboxistaurin or CGP41251 is reno-protective in rodent models of T1D (STZ) and/or T2D (db/db model)^16,26,29^. Importantly, however, some PKC family members have protective roles in the kidney: mice deficient in PKCε develop spontaneous kidney disease^30^, and PKCs ζ and ι/λ are required for the maintenance of podocyte polarity and function^31,32^. Therefore, selective inhibition of PKCα and PKCβ, one that spares other PKC family members, constitutes a promising therapeutic strategy to prevent diabetes-induced kidney injury.

Here we evaluate an orally bioavailable and selective PKCα/β dual inhibitor, Cmpd 1^33,34^, in the ZSF1 rat model of T2D. ZSF1 rats are obtained from a cross of two strains heterozygous for leptin receptor mutations^35^. F1 progeny that are not homozygous for leptin receptor deficiency are normophagic and do not develop disease (ZSF1 lean rats), whereas F1 progeny that are homozygous for leptin receptor deficiency are hyperphagic and become obese on high carbohydrate diet^35–40^. ZSF1 obese rats are one of relatively few translationally relevant rodent models of T2D characterized by DN progression to ESRD, with death resulting at 45–50 weeks of age. Therapeutic administration of Cmpd 1 was reno-protective, however the effect was indirect and due to an unexpected and striking effect of a blunted hyperphagic response. This was demonstrated through the inclusion of a pair feeding group in the present study. However, above and beyond this effect on food intake, Cmpd 1 lowered overall body weight, reduced liver vacuolation, and reduced the mass, size and inflammation in inguinal white adipose tissue (iWAT) in ZSF1 obese animals in a manner that was not recapitulated simply by pair feeding.

## Results

### Cmpd 1 is a potent, ATP-competitive inhibitor of PKCα and PKCβ

**Figure 1:**
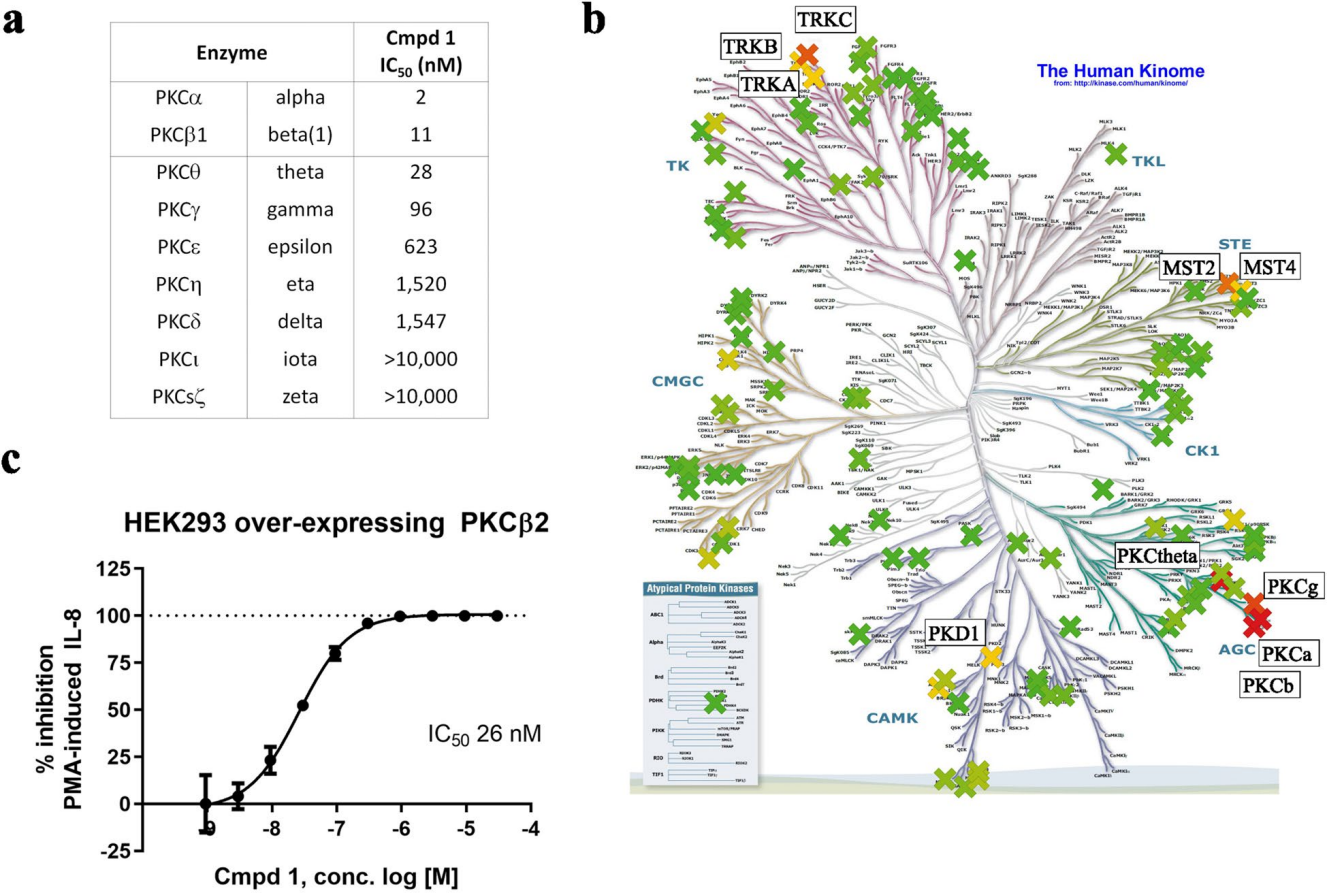
Compound 1 (Cmpd 1; Cmpd A in Ref. 34) is a potent, cell-permeable dual inhibitor of PKCα and PKCβ with a good selectivity profile. (**a**) Half-maximal inhibitory concentration (IC_50_) of Cmpd 1 against PKC family members in recombinant enzyme assays. (**b**) Kinome tree of selectivity profiling of Cmpd 1 against a panel of 119 kinases. The original kinome tree was taken from Ref.^68^. The percent inhibition by Cmpd 1 at 1 micromolar (µM) testing concentration is color coded, ranging from 0% inhibition (darker green) to 100% inhibition (darker red), with 50% inhibition displayed in yellow. All kinases with > 50% inhibition are labeled. (**c**) Percent inhibition by Cmpd 1 in a cell-based assay system using HEK293 cells overexpressing PKCβ2 that produce IL-8 in response to the PKC activator phorbol 12-myristate 13-acetate (PMA; representative experiment shown). Error bars here and in subsequent figures are −/+ standard deviation (SD).

Cmpd 1 is an example of a 3-Aminopyrrolo[3,4-c]pyrazole-5(1H,4H,6H)carboxaldehyde ATP-competitive inhibitor^33^, and is “Cmpd A” (5-[(2S,5R)-2,5-dimethyl-4-(tetrahydro-2H-pyran-4-ylmethyl)piperazin-l-yl]carbonyl-*N*-(5-fluoro-2-methylpyrimidin-4-yl)-6, 6-dimethyl-1,4, 5, 6-tetrahydropyrrolo[3,4-c]pyrazol-3-amine) in Ref.^34^. Figure 1a is a summary of the half maximal inhibitory concentration (IC_50_) of Cmpd 1 in enzymatic assays against PKC family proteins. IC_50_ values for PKCα and PKCβ were 2 and 11 nanomolar (nM), respectively. Cmpd 1 had moderate activity against some other PKC family members, notably PKCθ (IC_50_ 28 nM) and PKCγ (IC_50_ 96 nM). Selectivity against all other PKC family members was greater than 40-fold, and kinome profiling revealed good selectivity against a panel of 119 kinases (Fig. 1b). In a cell-based setting, Cmpd 1 inhibited the production of IL-8 in response to the DAG mimic and PKC activator phorbol 12-myristate 13-acetate (PMA) with an IC_50_ value of 26 nM (Fig. 1c). In summary, Cmpd 1 is a potent and cell-permeable PKCα/β dual inhibitor with reasonably good selectivity over other PKC family members and the kinome overall.

### Cmpd 1 inhibits PKC activity using source material from glomeruli

**Figure 2:**
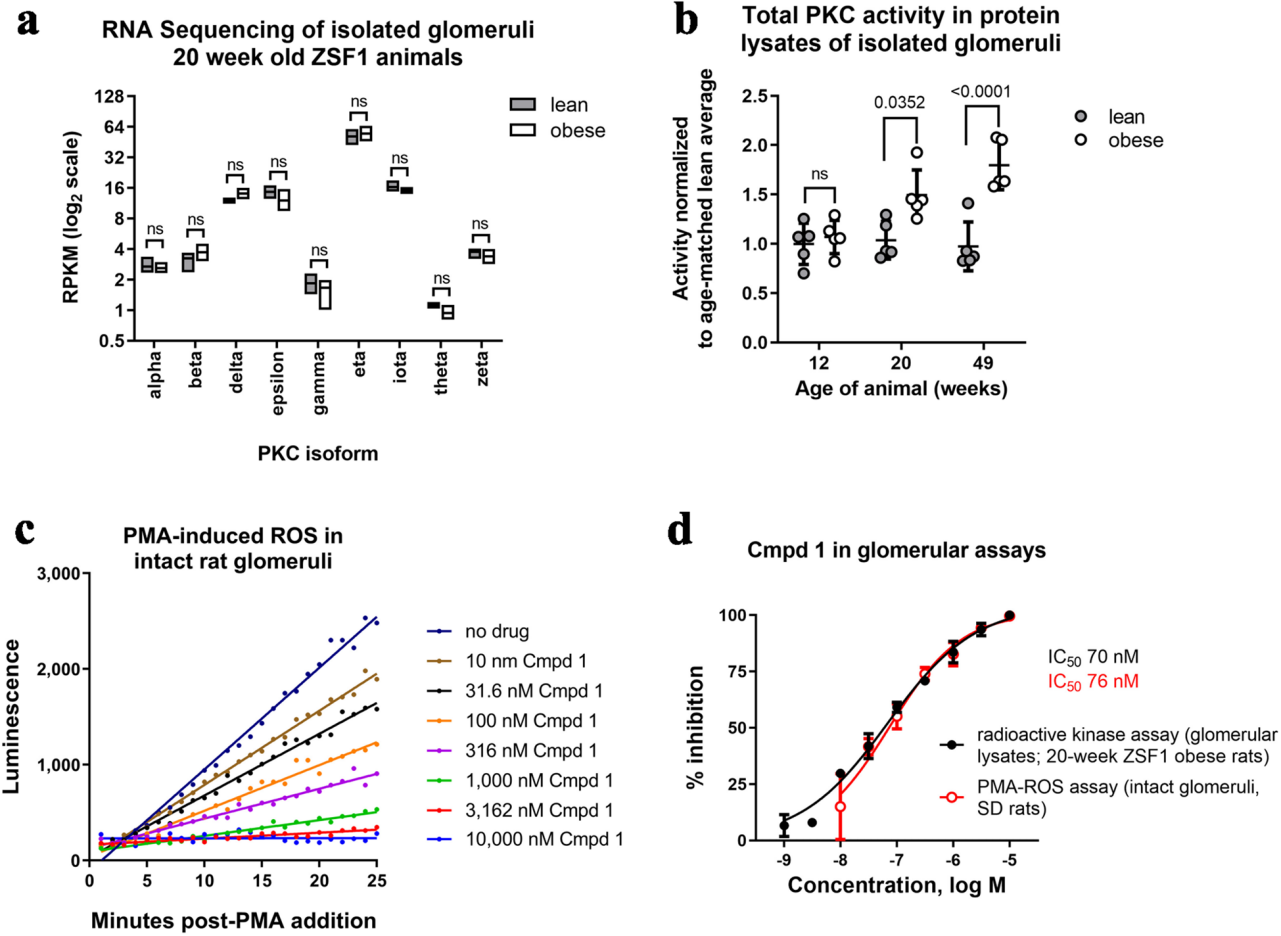
Cmpd 1 inhibits PKC in vitro in PKC activity assays using glomerular source material. Here and elsewhere, ZSF1 lean and obese rats were obtained at 8–9 weeks of age at which time they were switched to high carbohydrate Purina 5008 chow (27% kcal protein, 17% kcal fat, 57% kcal carboydrates). (**a**) mRNA abundance of PKC isoforms by RNA sequencing of isolated glomeruli from 20-week old ZSF1 lean or ZSF1 obese rats (n = 5 each). RPKM = reads per kilobase per million reads. (**b**) Pan-PKC activity in protein lysates made from glomeruli isolated from ZSF1 lean or obese rats at 12, 20, and 49 weeks of age (n = 5 each). Activity was measured in an in vitro radioactive kinase assay using a pan-PKC pseudo-substrate peptide (RFARKGSLRQKNV). (**c**) Luminol luminescence assay for the kinetics of ROS production following PMA stimulation of intact glomeruli isolated from SD rats in the presence of increasing amounts of Cmpd 1 (representative experiment shown). (**d**) Inhibition profiles of Cmpd 1 in a radioactive kinase assay with glomerular lysates from 20-week-old ZSF1 obese animals (average of n = 2 lysates), and in the representative PMA-ROS assay depicted in (**c**). Percent inhibition is normalized to no inhibitor (0%) versus 10 μM Cmpd 1 (100%). Statistical values, here and in subsequent figures, are by Anova Tukey’s multiple comparisons tests where “ns” = not significant.

The roles of PKCα and PKCβ in DN have been attributed to functions in podocytes and mesangial cells, respectively, within the kidney glomerulus^23–28^. All PKC isoforms were detectable in glomeruli isolated from ZSF1 rats based on our previously published transcriptomics analysis^37^, and there was no statistical difference between ZSF1 lean and obese rats for any PKC family member by mRNA abundance (Fig. 2a).

To measure PKC activity and Cmpd 1 pharmacology in glomerular tissue, we performed in vitro assays with glomeruli isolated by serially sieving minced kidney cortex. In the first assay, protein lysates were generated from glomeruli isolated from ZSF1 lean or ZSF1 obese rats at 12, 20, and 49 weeks of age. PKC activity in these lysates was determined in a radioactive kinase assay using a pan-PKC pseudo-substrate peptide. By this assay, PKC activity was similar in ZSF1 lean and ZSF1 obese rats at 12 weeks of age but was elevated in ZSF1 obese rats at 20 and 49 weeks of age (Fig. 2b). In a second assay, glomeruli from Sprague Dawley (SD) rats were isolated, left intact, and the kinetics of ROS production following PMA treatment was measured by Luminol luminescence^14,41^ (Fig. 2c). Based on the change in velocity of ROS production, an IC_50_ value of 76 nM was obtained (Fig. 2d; red line). Similarly, Cmpd 1 inhibited PKC activity when titrated into the radioactive kinase assay using glomerular lysates from 20-week old ZSF1 obese rats with an IC_50_ value of 70 nM (Fig. 2d; black line). Although a limitation of both assays is that they do not distinguish which PKC isoforms are responsible for the measured response, they indicate that Cmpd 1 can inhibit glomerular PKC activity that is intrinsic (radioactive kinase assay in lysates from 20-week old ZSF1 obese rat glomeruli) or induced (PMA-induced ROS in intact SD rat glomeruli) with double-digit nM potency.

### Cmpd 1 reduces hyperphagia and body weight in ZSF1 obese rats

**Figure 3:**
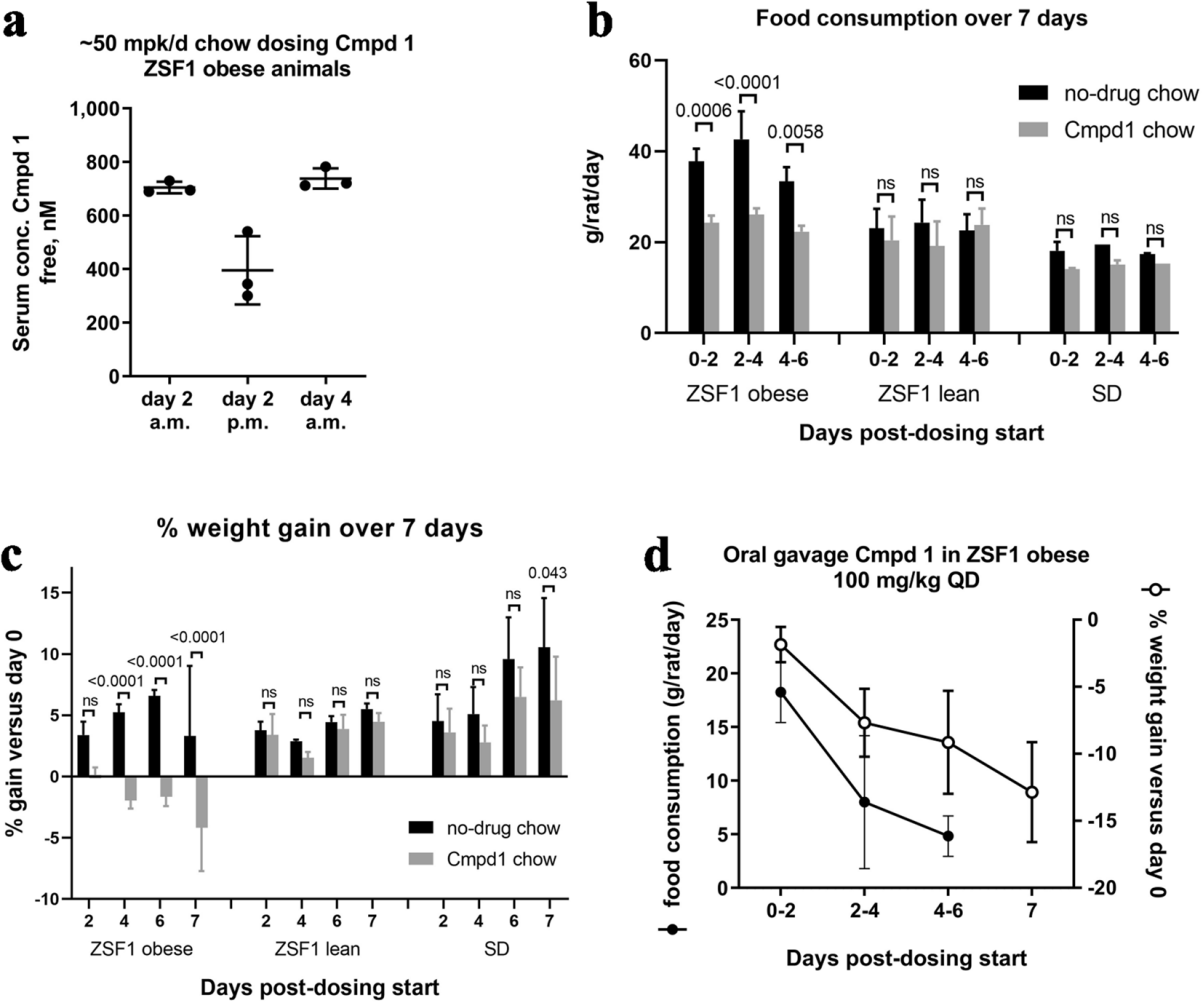
Cmpd 1 administration decreases food consumption and causes weight loss in ZSF1 obese animals. (**a**) Results from 4 day pharmacokinetic (PK) study for free (i.e., corrected for plasma protein binding) serum drug concentrations in the morning (9 a.m.) and late afternoon (4 p.m.) with chow dosing of Cmpd 1. Estimated food consumption (**b**) and % weight gain (**c**) over 7 days for ZSF1 obese, ZSF1 lean, or SD rats fed either no-drug chow or Cmpd 1 chow (n = 3 animals for each ZSF1 group; n = 2 animals for SD group). (d) Average food consumption of no-drug chow (left y-axis; closed circles) and % weight gain (right y-axis; open circles) of ZSF1 obese rats dosed daily with 100 mpk/day Cmpd 1 by oral gavage (n = 5 animals). Weights were measured on days 2, 4, 6, and 7.

In preparation for a long-term efficacy study we evaluated Cmpd 1 administration to ZSF1 obese rats through drug formulation in chow. Initial pharmacokinetic (PK) studies supported the use of a formulation to deliver approximately 50 milligram (mg) of Cmpd 1 per kilogram body weight (mpk) per day (50 mpk/day, or 0.744 g Cmpd 1 per kg chow). Results from a 4-day PK study with ZSF1 obese rats under this dosing regimen are shown in Fig. 3a. In the morning and evening respectively, free drug concentrations in serum were roughly 700 nM and 400 nM consistent with higher feeding activity at night. Based on enzyme and cell-based potencies for Cmpd 1, these concentrations were estimated to be sufficient to approach or exceed IC_90_ concentrations against both PKCα and PKCβ throughout the course of the day. Consistently, profiling kinase occupancy of spleens of Cmpd 1 chow-dosed ZSF1 obese animals using a probe-based chemoproteomics approach indicated > 90% occupancy of only PKCα/β from over 200 detectable kinases (Supplemental Fig. 1).

During these studies we noticed that ZSF1 obese rats appeared to consume less chow if it was formulated with Cmpd 1. To investigate this, we carried out a 7-day study measuring food consumption and body weight of ZSF1 obese rats provided Cmpd 1 chow versus regular (no-drug) chow. ZSF1 lean rats and SD rats were included for comparison. Food intake was estimated from periodic measurements of chow provided versus chow remaining in the food hopper. Over the study period, ZSF1 obese rats consumed an average of 38 grams (g) per day (g/day) no-drug chow; this value was 24 g/day for Cmpd 1 chow (a 37% reduction; Fig. 3b). By comparison, ZSF1 lean and SD rats consumed an average of 23 g/day and 18 g/day of no-drug chow, respectively, indicating that the reduced intake of Cmpd 1 chow by ZSF1 rats did not drop below normophagic levels. This effect of Cmpd 1 chow was considerably more pronounced in ZSF1 obese rats than in ZSF1 lean or SD rats, although a trend was also evident in these groups (Fig. 3b). ZSF1 obese rats fed Cmpd 1 chow also lost weight over the 7-day study period, whereas ZSF1 lean and SD rats did not (Fig. 3c).

To address the possibility that reduced food intake was an artifact, for example due to a potentially unpleasant taste of Cmpd 1, we evaluated ZSF1 obese rats administered Cmpd 1 daily by oral gavage (100 mpk daily; Fig. 3d). Over 7 days, both food consumption and animal body weights decreased. Thus, in 7-day studies, administration of Cmpd 1 through either chow or oral gavage blunted food intake and reduced the body weights of ZSF1 obese rats. Moreover, these effects were considerably more pronounced in ZSF1 obese rats than in ZSF1 lean or SD rats.

### 10-week efficacy study design

**Figure 4:**
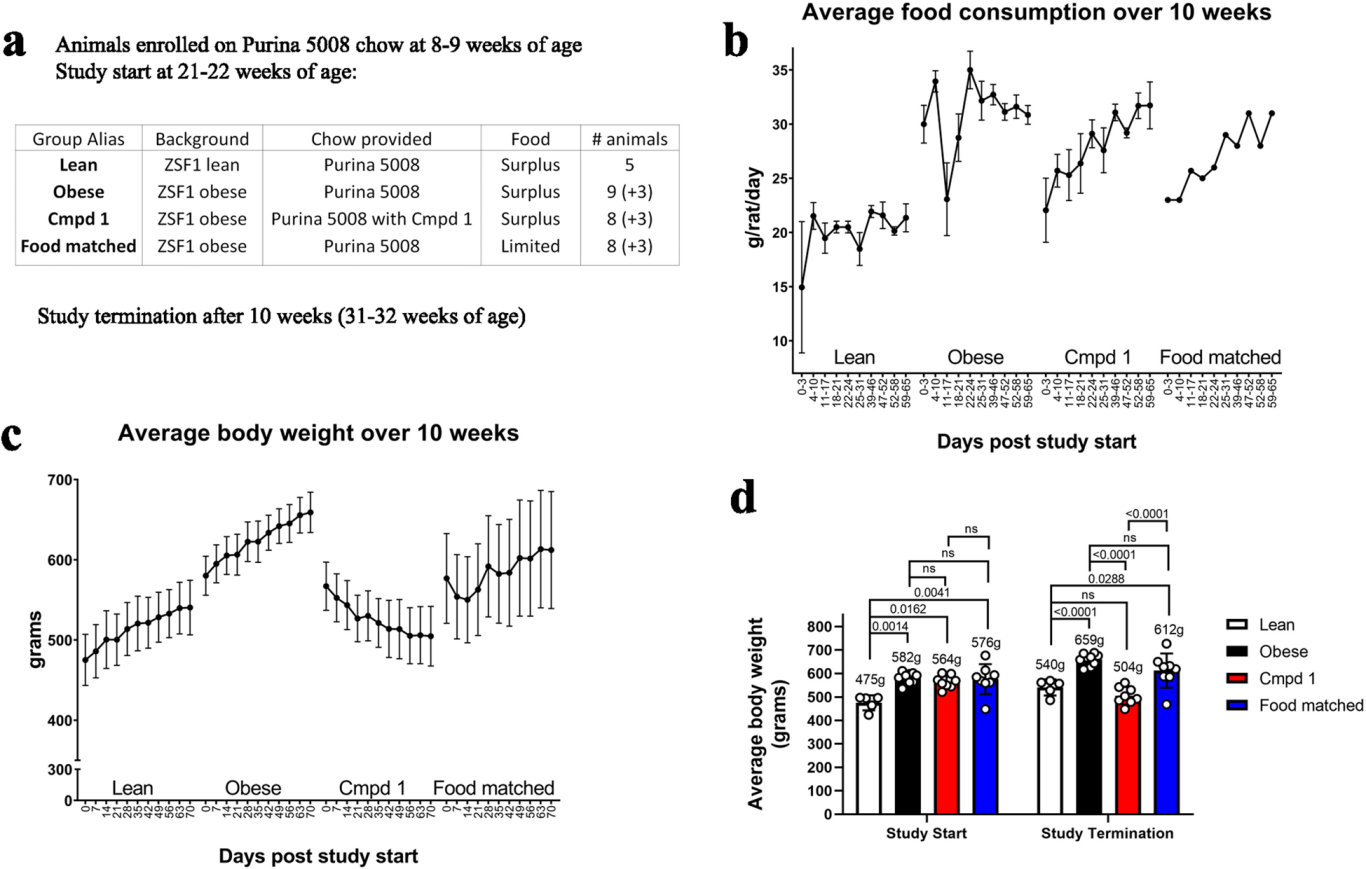
Study groups, food consumption, and body weight over the course of a 10-week Cmpd 1 administration study in ZSF1 obese rats. Animals were 21–22 weeks of age at study start. All groups were maintained on normal Purina 5008 chow for the 10-week study except the Cmpd 1 group, which was provided Purina 5008 chow formulated with 0.744 g Cmpd 1 per kg chow (approximatley 50 mpk/day Cmpd 1 administration; **a**). The amount of food provided each week to the Food matched group was determined by the estimated food consumption by the Cmpd 1 group the previous week. For all three ZSF1 obese groups, 3 animals were taken down at study mid-point for an interim analysis. (**b**) Average food consumption for all animals over the study duration as estimated by amount of food provided versus food remaining in the hopper for the indicated intervals. (**c**) Average body weight over the study duration for all animals. (**d**) Average body weights at study start and study termination (interim animals excluded), with average weights indicated numerically above the bars.

Based on these findings and previous experience with the model, we designed a therapeutic intervention study to evaluate chow dosing of Cmpd 1 for 10 weeks initiating in 21–22-week-old ZSF1 obese rats. We included a no-drug comparator group for which food intake was restricted to approximately match that of Cmpd 1-dosed animals. To achieve this, the estimated amount of Cmpd 1 chow consumed each week was monitored and this amount of no-drug chow was placed in the food hopper the following week for the comparator group. As a result, food intake amounts for these two groups were staggered by one week. A summary of the study groups is provided in Fig. 4a. Five age matched ZSF1 lean rats were included as a control (*Lean*). There were three groups of ZSF1 obese rats: a group provided no-drug chow (*Obese*), a group provided Cmpd 1 chow (*Cmpd 1*), and a group with estimated weekly food intake to match that of the Cmpd 1 group the previous week (*Food matched*). These three groups of ZSF1 obese rats consisted of 11 or 12 animals at study start, three of which were sacrificed at study mid-point for interim analyses. Food consumption and body weight were monitored weekly and non-fasting blood was drawn for analysis at 0, 5, and 10 weeks. Animals were placed in metabolic cages at 0, 5, and 10 weeks for renal function tests and sacrificed after the 10-week study duration, corresponding to approximately 32 weeks of age, for terminal histological and molecular analyses. All subsequent analyses include all data for all available animals unless otherwise noted.

### Food consumption and animal body weights over study duration

As anticipated, the Cmpd 1 group displayed an altered food intake pattern. In the first three days, the estimated average food consumption by this group was 22 g/day, down from 30 g/day in the Obese group (a 27% reduction; Fig. 4b). By comparison, the Lean group consumed an average of 15 g/day in this period. Food consumption in the Cmpd 1 group gradually normalized over the 10-week study duration, and by study termination these animals consumed an estimated 31 g/day compared to 32 g/day by the Obese group. However, despite this normalization, and in clear contrast to the Food matched group, the Cmpd 1 group steadily lost weight over the entire study duration (Fig. 4c). Indeed, at study termination, Cmpd 1 animals were on average approximately 7% less heavy, and not statistically different, than Lean animals (504 ± 35 g, versus 540 ± 30 g; Fig. 4d), despite consuming progressively higher amounts of food. In summary, the effect of Cmpd 1 in suppressing the hyperphagia of ZSF1 obese rats was recapitulated at the outset of this study but dissipated over time, presumably due to a compensatory increase in food intake as these animals lost weight.

### Non-fasting serum parameters

**Figure 5:**
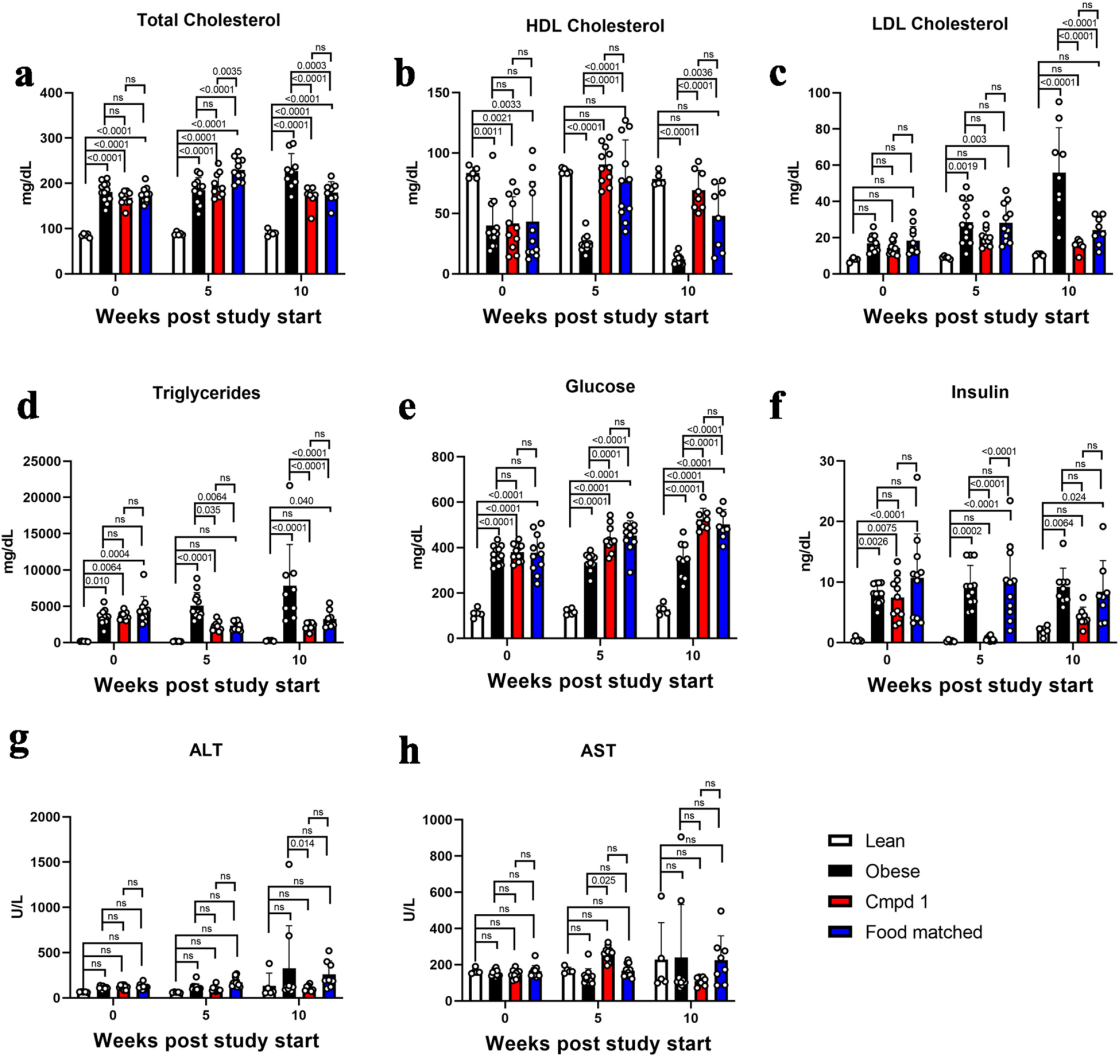
Non-fasting serum measurements at 0, 5, and 10 weeks study duration. (**a**) Serum total cholesterol, (**b**) HDL cholesterol, (**c**) LDL cholesterol, (**d**) triglycerides, (**e**) glucose, (**f**) insulin, (**g**) ALT, and (**h**) AST levels. Non-fasting conditions were used to minimize disruptions to feeding behavior during the study and are a limitation of these measurements.

We profiled serum chemistry at intervals over the study duration. Non-fasting conditions were used to minimize the impact of feeding disruptions on study outcomes, and are a significant limitation of these serum measurements. None-the-less, the data are provided here. ZSF1 obese rats displayed multiple features of metabolic syndrome at the study start age of 21–22 weeks, including elevated total cholesterol, reduced HDL cholesterol, elevated triglycerides, elevated glucose, and elevated insulin (Fig. 5a–f). Both Cmpd 1 and Food matched groups displayed a generally improved metabolic profile over the Obese group, in particular an improved cholesterol and triglyceride profile. Glucose levels for both groups were elevated. For most measurements, the Cmpd 1 and Food matched groups were not markedly different. A notable exception, however, was seen in insulin levels. While both Obese and Food matched groups displayed persistent hyperinsulinemia, Cmpd 1 animals appeared to have a reduction in insulin levels that was prominent at the 5 week timepoint however less evident at the 10 week timepoint. While the reasons for this are presently unclear, nonetheless by example, at the week 5 study midpoint the average insulin measurements for the various groups were as follows: 0.26 (± 0.16), 9.3 (± 3.5), 0.62 (± 0.35), and 9.9 (± 6.2) ng/mL for Lean, Obese, Cmpd 1, and Food matched groups respectively. Liver enzymes were not appreciably elevated in Obese animals (ALT and AST; Fig. 5g, h), indicating that liver function is not significantly impaired in this model setting. There were, however, statistically significant differences between Obese and Cmpd 1 animals in ALT at 10 weeks (reduced in Cmpd 1 group). There was also a statistically significant difference in AST at 5 weeks (increased in Cmpd 1 group). This difference was not evident at 10 weeks, and there was no statistical significance between Lean and Obese animals or between Lean and Cmpd 1 animals at any timepoint for either ALT or AST, possibly owing to the smaller number of animals in the Lean group.

### Cmpd 1 halts DN progression indirectly through its effect on food intake

**Figure 6:**
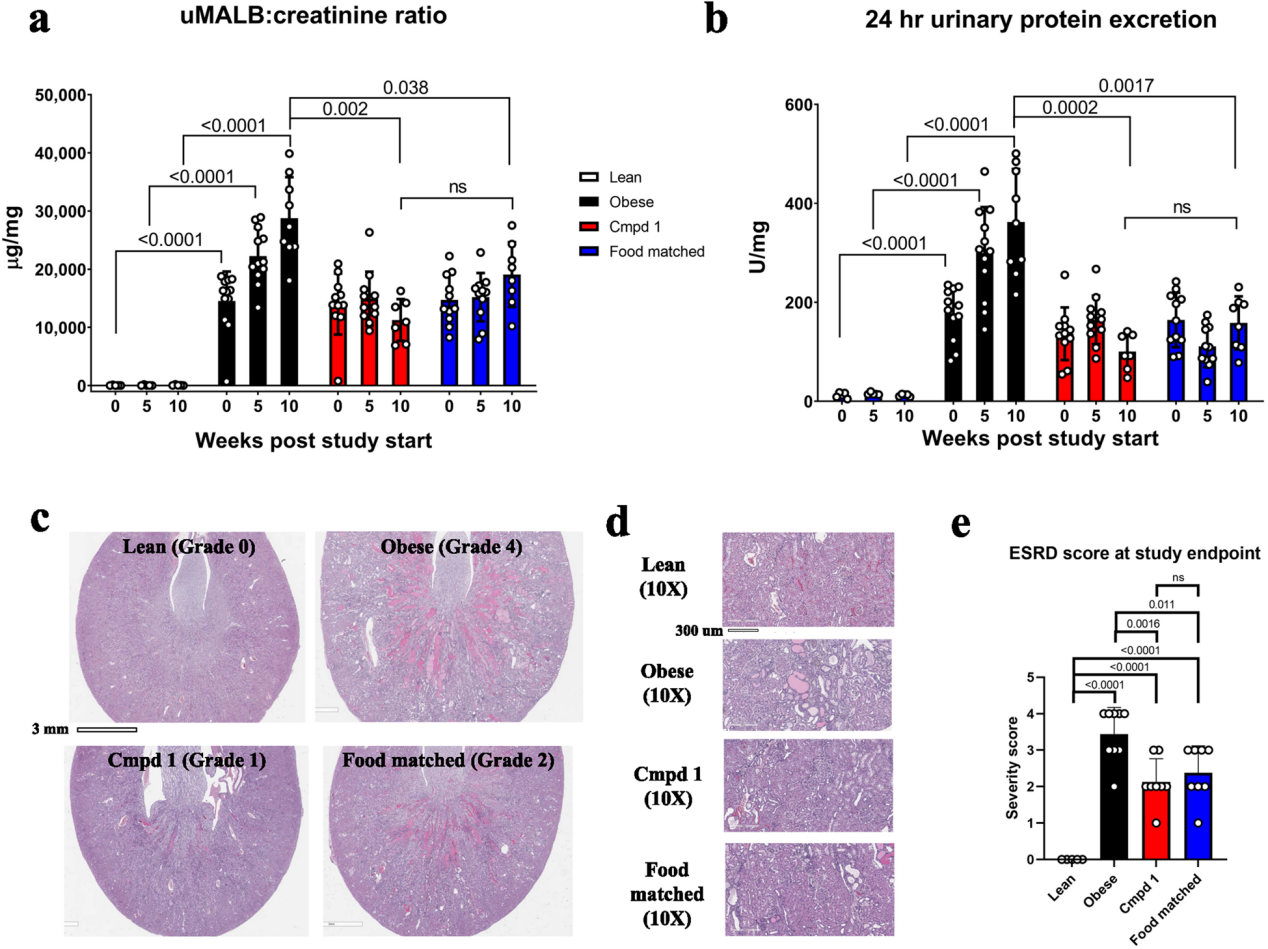
Cmpd 1 halts renal function impairment but does so indirectly through its effect on food intake. (**a**) Urinary microalbumin (uMALB) to creatinine ratio and (**b**) 24 h urinary protein excretion at study start, midpoint, and endpoint. We note that a data point is missing for one animal in the Cmpd 1 group at 10 weeks due to lack of urination by this animal in the metabolic cage. (**c**) Representative images of microscopic changes in the kidneys at study endpoint. Images were taken at 2× magnification and selected to illustrate a range of histopathological severity grades (described below). 10× magnifications are shown in (**d**). (**e**) End stage renal disease (ESRD) score following blinded analysis by a board-certified veterinary pathologist. A severity score of 1–5 (minimal to severe) was assigned using the following criteria: Grade 1 (minimal): < 10% of parenchyma affected, few glomeruli affected and no Bowman’s capsule thickening with only few protein casts and basophilic tubules observed; Grade 2 (mild): 10–25% of renal parenchyma affected, scattered thickening of Bowman’s capsule, +/− synechiation; Grade 3 (moderate): 26–50% of renal parenchyma affected, glomerular and tubulointerstitial changes present; Grade 4 (marked): 51–75% of renal parenchyma affected, glomerular and tubulointerstitial changes present; Grade 5 (severe): > 75% of renal parenchyma affected, glomerular and tubulointerstitial changes present.

Obese animals had renal impairment at their study start age of 21–22 weeks, with approximately 300-fold greater urinary microalbumin to creatinine (uMALB:creatinine) ratio than Lean animals (14,520 ± 4866 µg/mg versus 45 ± 42 µg/mg; Fig. 6a). This impairment progressively worsened over the 10-week study period as evidenced by increasing uMALB:creatinine ratio and 24-h urinary protein excretion in the Obese group (Fig. 6a, b). This progressive decline in renal function was virtually halted in the Cmpd 1 and Food matched groups.

By histology, microscopic findings at study termination in the kidneys in Obese animals included changes in the glomeruli and tubulointerstitial compartments (Fig. 6c, d; Supplemental Fig. 2). In the glomeruli, global to segmental multifocal mesangial expansion was observed with variable infiltrate of inflammatory cells, with and without synechiation, and thickening of the Bowman’s capsule. Tubular changes included hyaline protein casts within mostly medullary and cortico-medullary tubules, basophilic cortical and medullary tubules, tubular dilatation, attenuated basophilic epithelial cells, basement membrane thickening, occasional tubular hypertrophy within the cortex, and rare scattered mineralization. Variable expansion of the interstitium could also be observed, with mixed inflammatory cells and loose fibrous connective tissue. In a blinded analysis, the severity of kidney pathology (ESRD score) was graded from 1 (minimal) to 5 (severe; Fig. 6e). By this analysis, ESRD scores at study termination were reduced comparably in both Cmpd 1 and Food matched groups, consistent with the urine biomarker analyses. We conclude that therapeutic dosing of Cmpd 1 halted but did not reverse renal function decline, and did so indirectly through its effects on food intake in ZSF1 obese rats. Overall, a composite rather than a single histological metric was responsible for driving group differences in ESRD scores, with Cmpd 1 and Food matched groups scoring indistinguishably across metrics (Supplemental Fig. 2). Although renal endpoint profiles trended slightly better in Cmpd 1 animals than in Food matched animals, this did not achieve statistical significance, and we also note that reduced food intake for the Food matched animals was shifted by 1 week relative to Cmpd 1 animals over the study duration. Thus, any potential reno-protective effect of PKCα/β inhibition specifically in the glomerular compartment was not overtly evident given the over-riding effect of altered food intake pattern in halting renal function decline in the model setting.

### Cmpd 1 directly affects adiposity of liver and iWAT in ZSF1 obese rats

**Figure 7:**
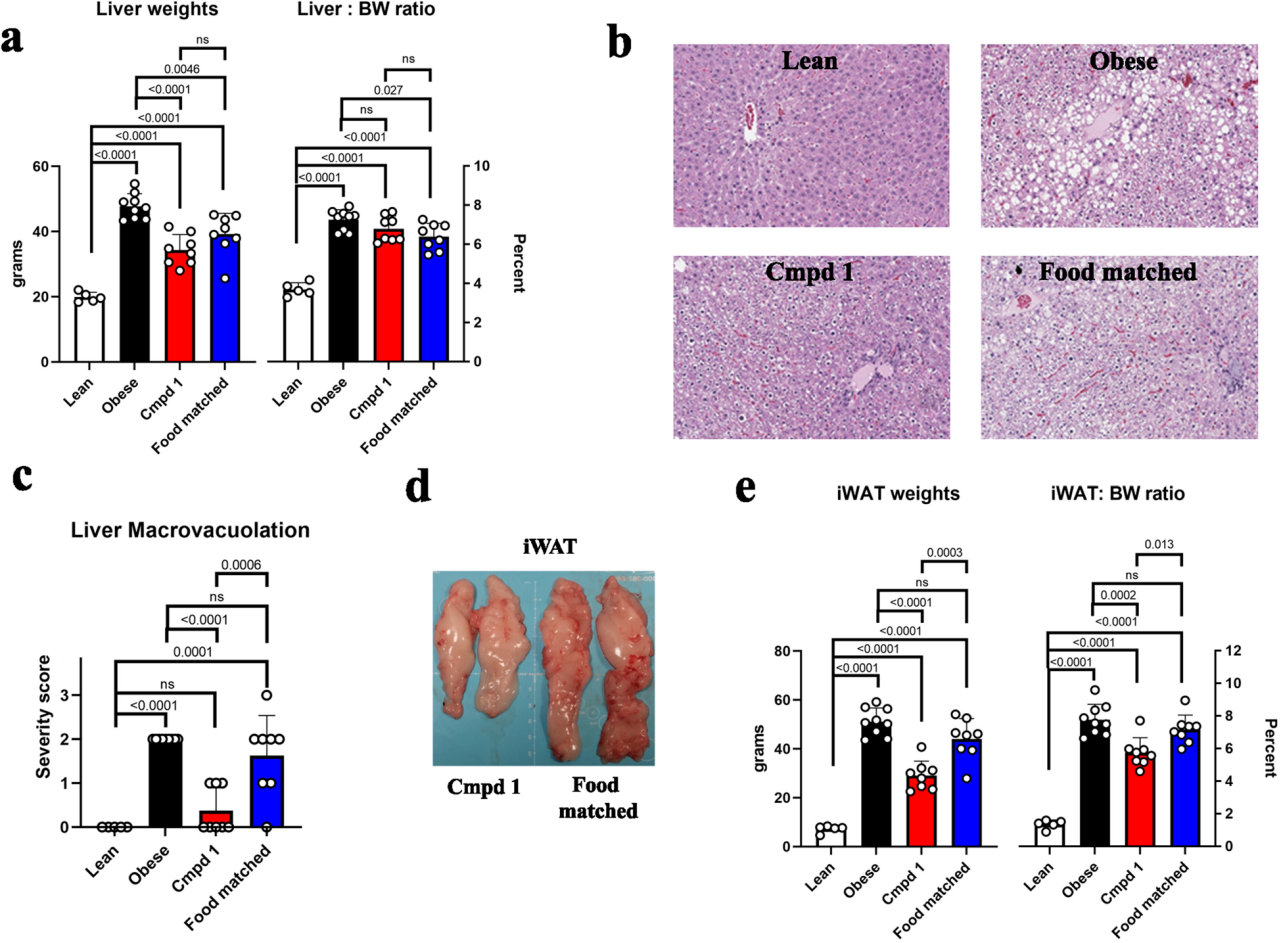
Cmpd 1 administration reduces liver macrovacuolation and the size of inguinal white adipose tissue (iWAT) in ZSF1 obese rats above and beyond its effect on food intake. (**a**) Liver weights and liver to body weight ratio (Liver:BW ratio) at study termination. (**b**) Representative microscopic findings in the liver at study termination, highlighting hepatocyte vacuolation. (**c**) Liver macrovaculation score of all animals at study termination based on blinded analysis by a board-certified veterinary pathologist assessed on HE-stained sections and assigned a severity score of 0–3 (no macrovacuolation to moderate macrovacuolation). (**d**) Image depicting size difference in iWAT of representative animals from Cmpd 1 and Food matched groups. (**e**) iWAT weights and iWAT:BW ratio at study termination.

We examined two other tissues relevant to the model and our observations: liver and inguinal white adipose tissue (iWAT). Cmpd 1 animals had reduced liver weights; however, this reduction primarily reflected differences in overall body weights (BW; liver to BW ratio; Fig. 7a). Histological analyses, in contrast, revealed differences in the livers across the groups. Obese animals had fatty livers as exemplified in the representative histology images in Fig. 7b. Microscopic findings included vacuolation, presumably from lipid accumulation, characterized as centrilobular to midzonal, and diffuse or multifocal macrovacuolation of hepatocytes with eccentric nuclei (consistent with lipid). To compare treatment groups, the severity score of liver macrovacuolation were assigned in blinded fashion and ranged from 0 (no macrovacuolation) to 3 (moderate macrovacuolation; Fig. 7c). Cmpd 1 reduced liver macrovacuolation scores in a manner not seen in the Food matched group: after 10-weeks of dosing, five of eight Cmpd 1 animals had liver macrovacuolation scores of 0 compared to only one of eight animals in the Food matched group. No appreciable necrosis was evident in the liver at study termination, and there was also no evidence of liver fibrosis by Picrosirius red staining (PSR; Supplemental Fig. 3). The model therefore appears to manifest features of simple steatosis but not of more advanced liver disease, with liver function largely unimpaired as evidenced by the lack elevation in AST or ALT (Fig. 5).

One striking find at necropsy was that the size of iWAT tissues was noticeably different across the groups, including between Cmpd 1 and Food matched animals (Fig. 7d). Cmpd 1 animals had substantially lower iWAT weights (29 ± 5.5 g) than Obese animals (51 ± 5.3 g) or Food matched animals (44 ± 7.8  g; Fig. 7e), although still considerably higher than iWAT weights in Lean animals (7.23 ± 1.32 g). Unlike with liver weights, differences in iWAT weights were not due to differences in overall body weights (iWAT to BW ratio; Fig. 7e). Taken together, Cmpd 1 had favorable effects on adipose tissue and overall adiposity in ZSF1 obese animals above and beyond its food intake effect, and in accordance with the body weight profiles over the study duration.

### Molecular and cellular changes in iWAT with Cmpd 1 administration

**Figure 8:**
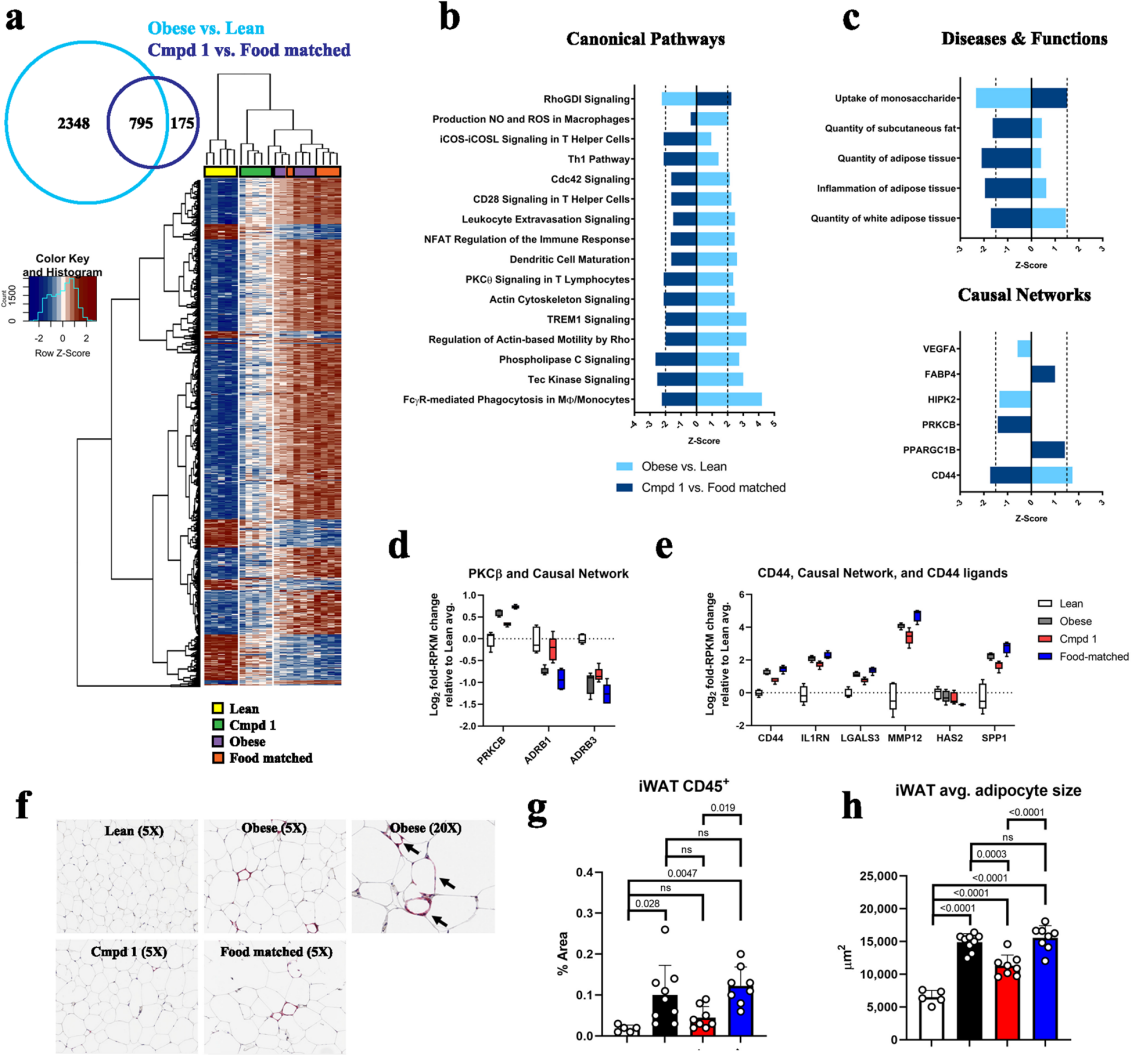
Transcriptomic and histological changes in iWAT with Cmpd 1 administration. (**a**) Venn diagram of differentially expressed genes (DEGs) in the two indicated contrasts. A heat map of unsupervised hierarchical clustering of the 795 DEGs present in both comparisons is shown. (**b**) Canonical pathways and (**c**) Diseases & Functions and Causal networks identified by Ingenuity Pathway Analysis (IPA) of these DEGs. Log_2_ fold-change relative to Lean average of the indicated transcripts for two of the identified Causal Networks, PRKCB (aka PKCβ; panel **d**) and CD44 (panel **e**). PKRCB was identified through changes in expression of ADRB1 and ADRB3. CD44 was identified through changes in expression of IL1RN, LGALS3, and MMP12. Two genes linked to CD44 ligands, HAS2 (hyaluron synthase 2) and SPP1 (osteopontin), are also shown. (**f**) Representative CD45 staining, in red, of iWAT tissue. A representative higher magnification image of an Obese animal is provided showing CD45^+^ cells (arrows) residing adjacent to and encircling adipocytes. (**g**) Quantitation of CD45^+^ cells in whole slides using digital image analysis; data represents percent of iWAT area that is reactive for CD45 staining. (**h**) Quantification of adipocyte size, in μm^2^, in iWAT by histology for all animals.

Given the dramatic impact of Cmpd 1 administration on iWAT this tissue was selected for more detailed molecular analysis. We performed bulk RNA sequencing of iWAT tissue from five randomly selected animals from each group. Differential expression analysis was conducted for two contrasts: Obese versus Lean groups (disease-associated changes), and Cmpd 1 versus Food matched groups (Cmpd 1-associated changes not due to changes in food intake). By this analysis, we identified 2523 disease-associated differentially expressed genes (DEGs), 970 Cmpd 1-associated DEGs, and 795 DEGs that were present in both contrasts (Fig. 8a). Hierarchical clustering by Euclidean distance of these 795 shared DEGs differentiated between Lean and Obese groups, and between Cmpd 1 and Food matched groups (Fig. 8a). A clear, albeit partial, recovery towards the Lean profile was observed in the Cmpd 1 group. Canonical Ingenuity Pathway Analysis (IPA) identified multiple pathways related to immune function as being elevated in Obese animals (Z-score greater than zero) and conversely inhibited in Cmpd 1 animals (Z-score less than zero; Fig. 8b). Further analysis using IPA Diseases & Functions annotations identified “quantity of adipose tissue”, “subcutaneous fat”, and “white adipose tissue” as being elevated with disease and inhibited by Cmpd 1, whereas “uptake of monosaccharide” was reduced with disease and rescued by Cmpd 1 (Fig. 8c). Causal network analysis identified several networks that were either disease-associated (VEGFA and HIPK2, both down) or Cmpd 1-associated (FABP4 and PRKCB aka PKCβ, both down; PPARGC1B, up), and only one network, CD44, that was reciprocally regulated in the two contrasts (Fig. 8c). The expression of PKCβ and its causal network genes identified by this analysis (the β-adrenergic receptors ADRB1 and ADRB2), and of the cell surface glycoprotein CD44 and its causal network genes (IL1RN, LGALS3, and MMP12), are provided in Fig. 8d and e. The expression of HAS2, which catalyzes the synthesis of the CD44 ligand hyaluronic acid, and SPP1 (aka Osteopontin), another ligand of CD44, are also shown in Fig. 8e.

A clear finding from the pathway analysis was a reduction in inflammatory signature in iWAT upon Cmpd 1 administration. To determine if this was due to reduced inflammatory cell numbers we performed immunohistochemistry using the pan-leukocyte marker CD45. CD45-positive (CD45^+^) cell numbers in iWAT increased with disease, with positive cells tending to reside adjacent to and encircling adipocytes (Fig. 8f). Average CD45^+^ cell staining area was lower in Cmpd 1 animals relative to Food matched animals (0.045 ± 0.025 percent, versus 0.122 ± 0.04 percent; Fig. 8g). Although variability within the Obese group precluded clear comparisons, an overall trend towards fewer CD45^+^ cells in iWAT was evident in the Cmpd 1 group: indeed, only the Cmpd1 group was not statistically different from the Lean group. This suggests that the effects on inflammatory pathways observed by molecular profiling were due, at least in part, to reduced inflammatory cell numbers in iWAT following Cmpd 1 administration. In addition, the histological analysis revealed that adipocytes assumed a morphology consistent with white fat and were increased in size in the Obese group, and that this effect was blunted in the Cmpd 1 group but not in the Food matched group (Fig. 8h). In summary, consistent with its macroscopic effect on the mass of iWAT tissue and overall animal body weights, Cmpd 1 had direct effects on inflammation and on adipocyte morphology and potentially also function in iWAT of ZSF1 obese rats.

## Discussion

Here we describe, to our knowledge, the first report of therapeutic dosing of a selective, dual small molecule inhibitor of PKCα and PKCβ in a rodent model of obesity-driven T2D characterized by progressive renal impairment. In pilot studies we observed that Cmpd 1 blunted the hyperphagia response of ZSF1 obese rats. We do not presently understand why. Animals such as ZSF1 obese rats that are deficient in leptin receptor signaling are hyperphagic likely due, at least in part, to an inability to reduce AMPK activity in the hypothalamus as a normal consequence of the satiety hormone leptin^42,43^. PKCs have been implicated in inhibiting AMPK, and in particular when DAG levels are elevated such as in conditions of hyperglycemia and hyperlipidemia^44^. Inhibition of hypothalamic PKCs, therefore, could in theory mimic leptin action. Although Cmpd 1 is not expected to pass the blood–brain barrier based on its structural features, we cannot exclude an effect of Cmpd 1 in the hypothalamus. In addition, potential changes to the blood–brain barrier under conditions of metabolic syndrome would need to be taken into consideration^45^.

PKCα, for which research has focused more on cardiovascular dysfunction, immune-mediated arterial thrombosis, and cancer, has not been previously linked to body weight or adipose tissue homeostasis^46^. However, previous work using PKCβ deficient mice both in a wild type background and in the background of leptin deficiency (ob/ob mice) revealed a clear role for PKCβ in adiposity and obesity^47–50^. In both wild type and ob/ob backgrounds, deletion of PKCβ led to reductions across adiposity, hepatic steatosis, and visceral and iWAT mass without a concomitant reduction in food consumption. While this latter observation is in apparent contrast the effects on hyperphagia we observe in our study, we note that food intake by Cmpd 1 animals was initially depressed but increased to normal levels by the 10 week time-point. Pharmacologically, Ruboxistaurin caused weight loss in (mRen-2)27 transgenic hypertensive rats but only when co-administered STZ, suggesting that the weight loss effect was linked to the T1D condition^51^. However, in another report, Ruboxistaurin prevented weight gain in animals administered the anti-psychotic drug clozapine but did so without impacting the slightly elevated food consumption patterns with clozapine administration^52^. Given the striking effect of Cmpd 1 on hyperphagia in the present study, and of Cmpd 1 and PKC inhibitors on body weight and adiposity in multiple pre-clinical contexts, future work should evaluate whether these phenomena are model-specific, rodent-specific, or more general in nature.

From RNA sequencing of iWAT we identified CD44 as a potentially important causal disease network that is induced by disease and reversed by Cmpd 1. CD44 is a cell-surface receptor for glycoproteins such as hyaluronan and secreted phosphoprotein 1 (SPP1; osteopontin) with roles in leukocyte migration and activation. Interestingly, in a high fat diet mouse model, CD44 blockade with a neutralizing antibody prevented obesity, reduced insulin resistance, and ameliorated adipose tissue inflammation^53^. While a link between PKC and CD44 is not conclusively known, it has been reported that activated PKCs phosphorylate CD44 to enable intracellular interactions between the CD44 cytosolic domain and the cytoskeleton, which is critical for CD44-induced cell motility^54^. Therefore, PKC inhibition could negatively impact CD44 action, resulting in reduced leukocyte accumulation. RNA sequencing revealed another network of potential interest, the β-adrenergic receptors ADRB1 and ADRB3, whose mRNA levels were downregulated with disease and rescued by Cmpd 1. This result is what implicated the PKCβ pathway in the causal network analysis. As ADRB1 and ADRB3 have been linked to promoting the proliferation of brown adipocytes and enhancing lipolysis^55,56^, this could constitute a potential mechanism for our findings with Cmpd 1 administration and PKC inhibition on iWAT size and adipocyte morphology, as has been proposed previously from studies using PKCβ knockout mice^48^.

Of relevance to the current study are previous studies in humans using Ruboxistaurin, also known as LY-333531. Ruboxistaurin has been shown to improve renal outcome in multiple rodent models of diabetes^15,29,57,58^. In large clinical trials, Ruboxistaurin at 32 mg/day for up to 3 years in diabetic individuals showed some benefit in diabetic eye complications, for which the trials were designed, but did not improve kidney function in post-hoc analyses^59–61^. We note, however, that although Ruboxistaurin is touted to be a PKCβ-selective inhibitor, it is rapidly metabolized to the much less-selective desmethylruboxistaurin in vivo^62–64^. In-so-far as multi-PKC inhibition but not pan-PKC or multi-kinase inhibition may be desirable to prevent diabetes-induced tissue damage, studies with Ruboxistaurin are consequently difficult to interpret with respect to whether the touted mechanism was selectively or suitably tested. Alternatively, in the absence of target engagement biomarkers, which can be difficult to obtain in the clinical setting, it is also possible that Ruboxistaurin did not achieve a target suppression profile in humans that we are able to achieve in our rodent studies.

We note several limitations of the current study. As with most studies using ATP-competitive inhibitors of kinases, off-target pharmacology is a concern. In addition, Cmpd 1 has activity against PKCθ, which has been linked to insulin sensitivity and obesity^65,66^. We attempted to minimize inhibition of kinases other than PKCα and PKCβ through chow dosing to avoid high peak-to-trough compound concentrations, but this limitation persists. An additional limitation is that we cannot assign observations from this study to PKCα inhibition, PKCβ inhibition, or inhibition of both; indeed, as the literature strongly suggests that many of our observations can be linked to PKCβ inhibition, it is presently unclear to what extent PKCα inhibition plays a role in any of our findings. A final and important limitation is that we were unable to evaluate a direct effect of Cmpd 1 administration on DN progression due to its food intake effect in this model setting.

Future studies should examine whether the effects we describe here for Cmpd 1 in the ZSF1 model setting translate to non-leptin models of obesity and whether they can be reasonably expected to extend to humans, in particular those afflicted by T2D. In addition, although obese ZSF1 rats display liver steatosis, they do not progress to nonalcoholic steatohepatitis (NASH)^67^. Given the rising incidence of NASH, for which T2D is a significant risk factor, and the beneficial effects we observe with respect to adiposity and inflammatory cell infiltration in adipose tissue, evaluation of Cmpd 1 or related PKC inhibitors in a pre-clinical NASH setting, and for obesity more generally, may be warranted.

## Methods

### Animals

Male ZSF1 (ZSF1-Lepr^fa^Lepr^cp^/Crl) lean rats (strain code #379) and ZSF1 obese rats (strain code #378) were obtained from Charles River Laboratories (Wilmington, MA). Male rats for all groups were obtained at 8–9 weeks of age at which time they were switched to high carbohydrate Purina 5008 chow (27% kcal protein, 17% kcal fat, 57% kcal carbohydrates). At 21–22 weeks of age they were then enrolled in the 10 week study. All groups were maintained on normal Purina 5008 chow for the 10 week study except the Cmpd 1 group, which was provided Purina 5008 chow formulated with 0.744g Cmpd 1 per kg chow (approximately 50 mpk/d Cmpd 1 administration; n = 5 Lean, n = 12 Obese, n = 11 Cmpd 1, n = 11 Food matched—see Fig. 4a). The amount of food provided to the Food matched group each week was determined by the estimated food consumption by the Cmpd 1 group the previous week. For all three ZSF1 obese groups, 3 animals were taken down at study mid-point for an interim analysis. All experimental protocols involving animals were reviewed and approved by the Pfizer Inc. Institutional Animal Care and Use Committee (IACUC). All experiments were performed in accordance with relevant guidelines and regulations, and all methods are reported in accordance with ARRIVE guidelines (https://arriveguidelines.org).

### In vitro kinase profiling

Half maximal inhibitory concentrations of Cmpd 1 were determined by Z’-Lyte biochemical assays (Life Technologies SelectScreen™ Kinase Profiling Services, Thermo Fisher Scientific). Kinome selectivity was determined at 1 μM Cmpd 1 concentration against a panel of 119 kinases. The original kinome tree in Fig. 1b was taken from Ref.^68^.

### In vitro assays

Glomeruli were isolated using an established protocol of serially sieving minced kidney cortex tissue through mesh filters of decreasing pore size^69^. For radioactive pan-PKC assays, glomeruli were lysed in a buffer consisting of 50 mM Tris–HCl pH 8, 5 mM EDTA, 5 mM EGTA, protease and phosphatase inhibitor cocktails, 1% 2-mercaptoethanol, and 0.028% n-dodecyl-β-d-maltopyranoside (Anatrace D310) by sonication using two 10 s pulses with a probe sonicator. Supernatants were collected following centrifugation at 4 °C at 20,200 × g for 10 min. A 25 μL aliquot was used in a 50 μL ^32^P-ATP pan-PKC radioactive kinase assay with the pan-PKC pseudo-substrate peptide RFARKGSLRQKNV. The reaction was incubated for 10 min at 37 °C prior to filter binding and scintillation counting. Data are reported as CPM incorporation per μg of total protein in the reaction, as determined using Pierce Coomassie Plus Protein Assay of the original lysate (Pierce 23236). Where indicated, the reaction was run in the presence of increasing amounts of Cmpd 1.

For the phorbol 12-myristate 13-acetate (PMA)-luminol assay, glomeruli isolated from PBS-perfused kidneys were diluted in an HBSS solution containing 1 mM luminol, 0.1 M HEPES, and 0.1% fatty-acid free BSA. Approximately 3000 glomeruli were plated per well in a 96-well white OPTI-plate (Perkin Elmer 6005290). The reaction was started by the addition of PMA to a final concentration of 16 μM in the absence or presence of increasing Cmpd 1. Luminol luminescence was read on an Envision plate reader in kinetic read mode.

For the HEK293 cell-based assay, HEK293 cells engineered to over-express full-length PKCβ2 were stimulated with 3 nM PMA in DMEM media supplemented with 0.5% FBS. 4-h cell culture supernatants were analyzed for IL-8 by MSD (Rockville, MD).

### Pharmacokinetic (PK) and food consumption studies

Three male 12-week old ZSF1 obese rats were fed control or 0.744 g/kg Cmpd 1 chow for 4 days. Food consumption was estimated by measuring food provided versus food remaining in the hopper. Blood was collected through tail vein from each animal at 9 a.m. and 4 p.m. 2 days after chow dosing started, and at 9 a.m. four days after chow dosing started, for PK determination. Plasma protein binding of Cmpd 1 was measured by equilibrium dialysis of the in vitro fraction unbound of Cmpd 1 at a concentration of 2 mM in plasma from pooled male and female CD-1 mice. Plasma was centrifuged and supernatant was analyzed by liquid chromatography–tandem mass spectrometry to detect Cmpd 1 (York Bioanalytical Solutions; York, UK).

To evaluate food consumption in different rat strains, cohorts of 10 male ZSF1 obese rats, 8 male ZSF1 lean littermate rats, and 10 age-matched male Sprague Dawley (SD) rats were divided into two groups and fed chow with or without 0.744 g/kg Cmpd 1 (Cmpd 1 chow) for 7 days.

In vivo *kinase profiling* Spleens from 22–23 week old ZSF1 obese male rats provided no drug Chow or Cmpd 1 chow (n = 2 each) were snap frozen, pulverized, and approximately 100–150 mg of each sample were sent to ActiveX for analysis using a probe-based chemoproteomics platform to measure the fraction of kinase bound versus unbound by an ATP-competitive inhibitor. A kinase bound by an ATP-competitive inhibitor such as Cmpd 1 will be less accessible to a covalent ATP probe, and will consequently have a reduced peak in quantitative mass spectrometry of the tryptic fragment corresponding to its active site pulled down by the covalent biotinylated ATP probe. Lysates from n = 2 animals for each group were combined for the analysis.

### 10-week efficacy study

Male ZSF1 obese rats that had been maintained on Purina 5008 chow were switched to Purina 5008 chow with or without 0.744 g/kg Cmpd 1 at 22 weeks of age. The study duration was 10 weeks. Estimated food consumption monitored weekly. An additional group of ZSF1 obese rats was included which received an amount of Purina 5008 chow to match the previous week’s estimated food consumption by the Cmpd 1 group. Five male ZSF1 lean littermates served as baseline controls. Intermediate blood samples were taken through tail vein. 24-h urine samples at weeks 0, 5 and 10 were collected from animals in metabolic cages. Animals were sacrificed by CO_2_ asphyxiation followed by blood collection via cardiac puncture and tissues fixation in 10% neutral buffered formalin for histology. Unfixed inguinal white adipose tissue was also collected at study termination for downstream molecular analyses.

### Serum cholesterol, HDL, LDL, triglyceride, glucose, and insulin assays

All assays except the insulin assay were run with the ADVIA 1800 Chemistry System (Siemens Medical Systems Diagnostics, Tarrytown, NY, USA). The serum insulin assay was run using a SpectraMax® Plus384 Absorbance Microplate Reader (Molecular Devices San Jose, CA, USA)***.*** Siemens Medical System assay kits 10376501, 10311891, 10335892, and 10335891 were used for cholesterol, low density lipoprotein (LDL)-cholesterol, triglycerides, and glucose, respectively. Roche HDL-C Reagent 3rd generation assay kit 4713257190 was used for high density lipoprotein (HDL)-cholesterol assay. For insulin, an ALPCO Diagnostics assay kit 80-INSMS-E01 was used.

### Histopathology

Samples of formalin-fixed, paraffin embedded (FFPE) liver and kidney were sectioned and stained for Hematoxylin and Eosin (HE) and evaluated microscopically by a board-certified veterinary pathologist blinded to the treatment groups. In the kidney, microscopic features were assigned an end stage renal disease (ESRD) severity score of 1–5 (minimal to severe) using the following criteria: Grade 1 (minimal): < 10% of parenchyma affected, few glomeruli affected and no Bowman’s capsule thickening with only few protein casts and basophilic tubules observed; Grade 2 (mild): 10–25% of renal parenchyma affected, there is scattered thickening of Bowman’s capsule, +/− synechiation; Grade 3 (moderate): 26–50% of renal parenchyma affected, glomerular and tubulointerstitial changes are present; Grade 4 (marked): 51–75% of renal parenchyma affected, glomerular and tubulointerstitial changes are present; Grade 5 (severe): > 75% of renal parenchyma affected, glomerular and tubulointerstitial changes are present. In the liver, hepatocellular vacuolation was assessed on HE-stained sections and assigned a severity score of 0–3 (no macrovacuolation to moderate macrovacuolation). FFPE inguinal white adipose tissue was sectioned and stained for HE and the pan-leukocyte marker CD45 was detected by anti-CD45 ab10558 (Abcam, Cambridge MA; 1:5000 dilution) and red chromogen on the Leica Bond Auto-stainer Bond-Rx using heat induced epitope retrieval 2—pH 9 (HIER 2) for 20 min. Positive controls consisted of banked FFPE rat lymph node and GALT. Whole-slide images of the immuno-stained sections were obtained with a Leica AT2 whole slide scanner (Leica Microsystems GmbH) for use in image analysis. CD45 immuno-positive areas were quantified using Definiens Tissue Studio image analysis software (Definiens AG, Germany) and quantitative data were reported as percentage of CD45 IHC stain vs total tissue section areas. For adipocyte size measurements, whole-slide images of the HE stained sections were obtained with a Leica AT2 scanner (Leica Microsystems GmbH) and adipocyte size was assessed by measuring cross sectional area (CSA) of adipocytes using newCAST software (Visiopharm, Broomfield, CO). Briefly, 10% meander sampling of the whole slide images were selected, point probe was used to identify adipocytes to be measured and nucleator probe was used to measure the CSA of the adipocytes. Data were reported as mean CSA of adipocytes in the section from each animal. HE and CD45-stained slides and quantitative image analysis data were analyzed and interpreted by a board-certified veterinary pathologist. Scanned whole slide images of PSR-stained liver sections were analyzed using custom Bayesian classification algorithms developed in Visiopharm software (V2022.09) to quantify PSR+ area relative to total liver area on the slide, yielding a % area PSR stain. PAS-positivity in renal glomeruli was quantified in whole slide images of PAS-stained kidney with Visiopharm using a custom AI tissue detection algorithm to delineate individual glomeruli followed by quantification of PAS-positive area in all glomeruli using a Bayesian classifier. Data are expressed as total PAS-positive area in glomeruli in each section, normalized to the total number of glomeruli on the slide as this avoided confounding differences in glomerulus size related to the disease process.

### RNA sequencing of inguinal white adipose tissue

Total RNA from inguinal white adipose tissue was isolated using RNeasy Plus Universal Kits (Qiagen 73404). Rat genome and Ensemble gene annotations were downloaded from Ensembl. The clean raw sequence reads in FASTQ format were analyzed using the QuickRNASeq pipeline, and reads were mapped to the rat reference genome (rn6) using STAR v2.4.0h. Uniquely mapped reads were counted towards individual genes using featureCounts. Genes that had no sequence reads mapped to them in 50% of samples were labeled as no or low expressed, and thus omitted from the differential expression analysis. This filtering step was included to reduce the number of false positives in the differential analysis. A gene count table was generated by featureCounts and the differential analysis was performed by R packages edgeR 3.10.2 and Limma/voom 3.22.10. Genes with a fold change greater than 1.5 or less than − 1.5 and adjusted p-value (Benjamini-Hochberg) less than 0.05 were reported as significantly differentially expressed genes (DEGs).

### Pathway analysis

Pathway analysis was performed using QIAGEN Ingenuity Pathway Analysis (IPA). Two distinct Core Analyses were run: Obese versus Lean, and Cmpd 1 versus Food matched. A p-value less than 0.05 was set as the significance threshold for enriched pathways. A z-score algorithm was applied to determine if an enriched pathway was up- or down-regulated based on the input DEGs. A further Comparison Analysis between the two Core Analyses was run, and pathway enrichment findings were filtered for opposing trend and z-score in Obese versus Lean and Cmpd 1 versus Food matched. The Canonical Pathways and Diseases and Functions reported are those with a non-zero z-score in both comparisons, and p-value less than 0.05 and absolute z-score of 2, in at least one Core Analysis. The reported causal network regulators were all those with network-bias corrected p-value less than 0.05 in either Core Analysis.

### Statistics

Where shown, all error bars designate minus/plus Standard Deviation. For all analyses excluding RNA sequencing analyses (see above), p-values were calculated using 1-way or 2-way Anova with Tukey’s multiple comparison test for pairwise comparisons. “ns” denotes non-significant between pairwise comparisons; where significance was obtained, a numerical p-value is provided.

### Supplementary Information

Supplementary Figures.

## Data Availability

All datasets on which the conclusions of the paper rely will be made available to readers upon request. The datasets generated and/or analyzed during the current study are available in the GEO repository (https://www.ncbi.nlm.nih.gov/geo/) under accession number GSE161261. For requests for data from this study, please contact Ken Dower (ken.dower@pfizer.com). All authors are or were full-time employees and shareholders of Pfizer Inc. at the time the work was conducted.

## Abbreviations

PKC: Protein kinase C
DN: Diabetic nephropathy
T2D: Type 2 diabetes
T1D: Type 1 diabetes
ESRD: End stage renal disease
DAG: Diacylglycerol
AGE: Advanced glycosylation end products
ROS: Reactive oxygen species
STZ: Streptozotocin
PMA: Phorbol 12-myristate 13-acetate
PK: Pharmacokinetic
IC_50_: Half maximal inhibitory concentration
RPKM: Reads per kilobase per million reads
iWAT: Inguinal white adipose tissue
DEG: Differentially expressed gene
IPA: Ingenuity Pathway Analysis
NASH: Nonalcoholic steatohepatitis
