# Supplementary material for: A protein kinase C α and β inhibitor blunts hyperphagia to halt renal function decline and reduces adiposity in a rat model of obesity-driven type 2 diabetes
Source: Sci Rep. 2023 Oct 7;13:16919. doi: 10.1038/s41598-023-43759-7 (PMC10560236; doi:10.1038/s41598-023-43759-7)
Supplement: Supplementary file 1 — Supplementary Figures. [file 41598_2023_43759_MOESM1_ESM.pdf]

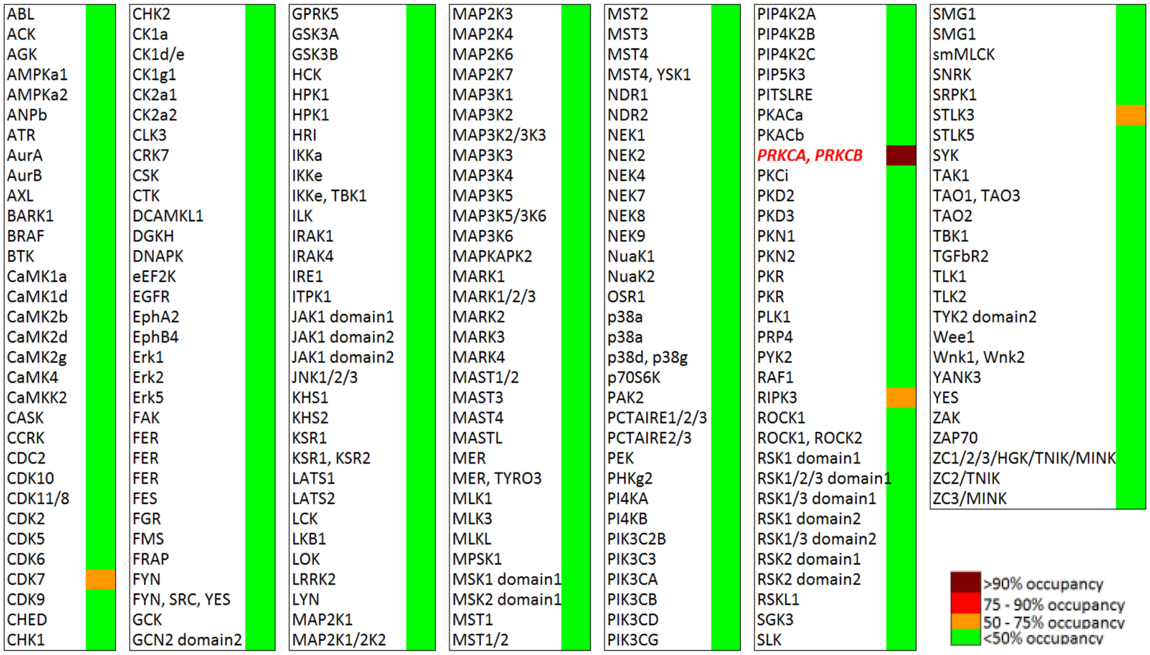

Supplemental Figure 1 Results of ActiveX kinase occupancy profiling of spleens from obese ZSF1 rats dosed 50 mpk/d Cmpd 1 chow compared to those receiving no drug. Briefly, this method quantitates the amount of kinase that can be pulled down by a covalent, biotinylated ATP probe under drug- and drug free-conditions. A kinase bound by an ATP-competitive inhibitor such as Cmpd 1is less accessible to probe, and will consequently have a reduced peak in quantitative mass spec when compared to drug-free conditions. Spleens from dosed animals were selected for analysis as they were expected to provid broad representation of the kinome. Using this method, over 200 kinases were detected in our samples. Among these, the only kinase with >90% occupancy with 50 mpk/d Cmpd 1 chow dosing was a trypic digest peptide corresponding to the active site of PKCα and PKCβ (PRKCA, PRKCB; the technology cannot distinguish one from the other as the tryptic digests of their active sites are identical). Among the remaining kinases detected, none had >75% occupancy, and three - CDK7, RIPK3, and STLK3 – had of 50-75% occupancy. Other PKC family members were not detected in the analysis; therefore, we cannot conclude that they were unaffected with 50 mpk/d dosing. Lysates from n=2 animals were combined for the analysis.

Wang et al Supplemental Figure 2

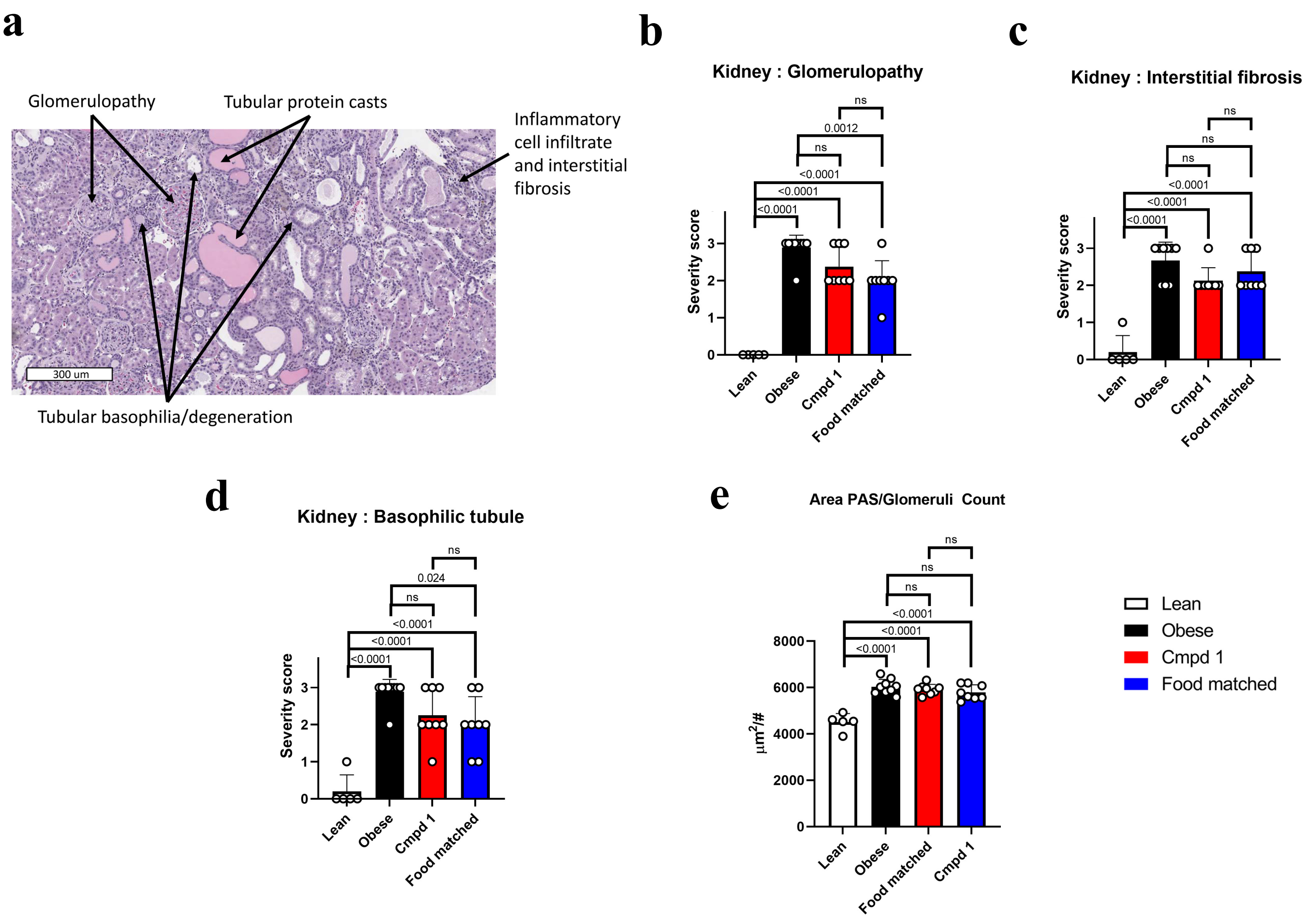

Supplemental Figure 2 Expanded renal analyses of study animals. (a) Annotated image of the representative ZSF1 obese animal image from Figure 6 with key disease-related findings marked. (b-d) Results of blinded severity scoring of individual histological features in the kidneys. Overall, no single histological metric was responsible for driving group differences in the final composite ESRD score, and Cmpd 1 and Food matched groups scored indistinguishably across metrics. (e) % area Periodic acid-Schiff (PAS) in all glomeruli divided by the number of glomeruli in the section to yield a PAS area/glomerulus ratio, which quantifies elements of PAS positive mesangial expansion and basement membrane thickening features included in the scoring "glomerulopathy."

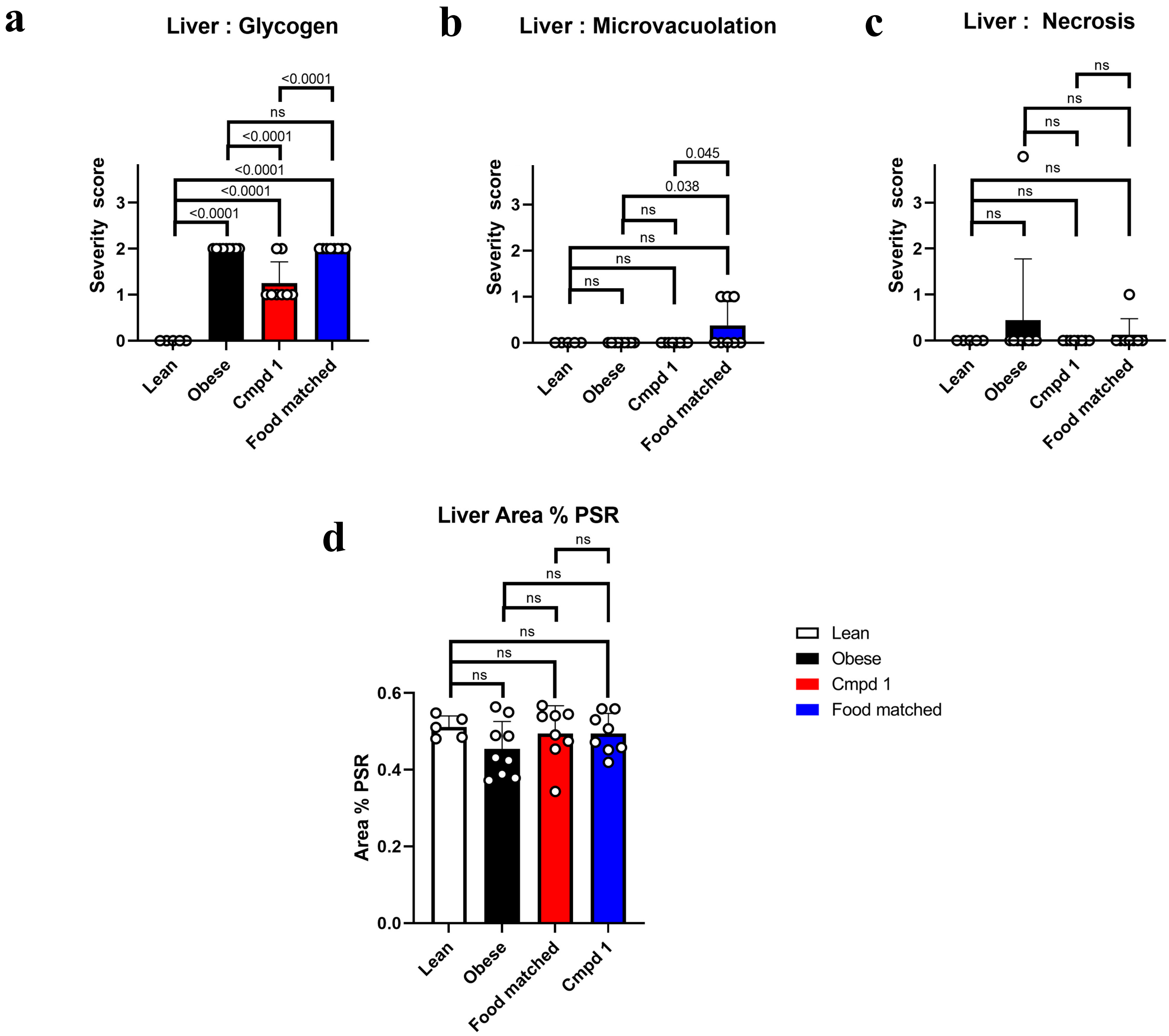

Supplemental Figure 3 Additional liver analyses of study animals. Microscopic severity scores by blinded analysis for (a) liver glycogen (PAS-stained sections), (b) microvacuolation, and (c) necrosis. (d) Liver fibrosis as determined by Picrosirius red (PSR) staining: despite the steatosis evident in the model, no appreciable necrosis or fibrosis was observed.
